# The effect of perceived organizational justice on workplace deviant behavior of new nurses: the role of emotional labor and psychological capital

**DOI:** 10.1186/s12912-024-01937-6

**Published:** 2024-04-28

**Authors:** Ran Meng, Zhe Jiang, Yue Su, Guangli Lu, Chaoran Chen

**Affiliations:** 1 Institute of Nursing and Health, School of Nursing and Health, Henan University, Kaifeng, China; 2 Institute of Business Administration, School of Business, Henan University, Kaifeng, China

**Keywords:** Perceived organizational justice, Emotional labor, Psychological capital, Workplace deviant behavior, Nurses

## Abstract

**Background:**

New nurses are prone to workplace deviant behavior in the constrained hospital environment, which will not only directly affect the safety of patients, but also reduce the work efficiency of nurses and bring negative results to the hospital. The purpose of this study was to investigate the relationship between perceived organizational justice, emotional labor, psychological capital, and workplace deviant behavior of new nurses.

**Methods:**

A cross-sectional study was used in this study. A survey was conducted in 5 hospitals in Henan Province, Chain from February to April 2023. The sample size was 546. The questionnaire included general information, perceived organizational justice scale, emotional labor scale, psychological capital scale, and workplace deviant behavior scale. SPSS 26.0 and PROCESS Macro were used for data analysis. PROCESS Model 4 and Model 14 were used to verify the model.

**Results:**

This study displays that perceived organizational justice was negatively correlated with emotional labor and workplace deviant behavior, and emotional labor was positively correlated with workplace deviant behavior. Meanwhile, emotional labor plays a partial mediating role between perceived organizational justice and workplace deviant behavior, accounting for 32.7% of the total effect. Moreover, the path of emotional labor on workplace deviant behavior is moderated by psychological capital.

**Conclusion:**

This study further understood the workplace deviant behavior of new nurses, and provided a new perspective for solving this problem. Nurse managers can reduce workplace deviant behavior by enhancing the perceived organizational justice and psychological capital of new nurses and improving emotional labor.

## Introduction

With the aggravation of the aging population, the demand for high-quality medical care services has intensified [1]. According to a report by the World Health Organization (WHO), there is still a shortage of more than 5.9 million nurses worldwide [2]. This means that the number of nurses is far from enough to meet the development of medicine. New nurses are the new human resources of the nursing industry, and their professional competencies directly influence the quality and development of nursing services [3]. New nurses refer to those who have worked for less than 1 year after graduation [4]. Faced with the role change from school to hospital and the complex clinical environment, new nurses generally lack confidence in clinical work and have different degrees of workplace maladjustment [5], resulting in interpersonal sensitivity and anxiety. The challenge extends further as they strive to strike a balance between their professional responsibilities and personal well-being [5], reducing their enthusiasm for clinical work and causing job burnout [6]. Moreover, new nurses often experience high-load nursing training before formally starting work, resulting in serious physical and mental load [3]. At the same time, the working conditions of many nurses and healthcare professionals around the world are also deteriorating due to COVID-19 [7], further affecting the initiative of nurses and leading to workplace deviant behavior.

Workplace deviant behavior is the voluntary behavior of organization members that violates important organizational norms and threatens the well-being of the organization and its members [8]. The workplace deviant behavior of nurses includes less harmful behaviors such as absenteeism, idleness, and lying, as well as more harmful behaviors such as spreading rumors, violating medical orders, knowing that medical orders are unreasonable but not followed, and violating nursing practices [9]. Nurses’ workplace deviant behavior will not only reduce their own work efficiency and work involvement [10, 11] but also affect their work performance. In addition, it also increases work pressure and turnover intention of colleagues, weakening job satisfaction, organizational commitment and happiness [12–14], and affects the stability of the nursing team [15]. On the other hand, deviant behavior will also affect occupational safety, cause medical safety accidents [16], and affect doctor-patient relationships. It can be seen that nursing staff’s deviant behavior is relatively common in clinical work, which not only causes negative effects on the team and members but also endangers the safety of patients and affects the process of disease rehabilitation.

Although previous studies have made contributions to the field of workplace deviant behavior, there are still some research gaps. Firstly, previous studies on workplace deviant behavior have mostly focused on inter-enterprise situations [17, 18], such as bank employees [14] and construction workers [11], while there is still relatively little research on the workplace deviant behavior of new nurses in medical settings. Secondly, there is a lack of research on the mechanisms of workplace deviant behavior. Existing research mostly focuses on the relationship between two variables, and there is insufficient research on the potential mechanisms of multiple variables and workplace deviant behavior, lacking effective theoretical support to guide clinical workers in taking measures. Finally, previous studies have mostly focused on the influence of leadership on workplace deviant behavior of nurses, with little exploration of the significance of nurse own psychological state on workplace deviant behavior, which may be a research gap. Nurses are often subjected to high-pressure work for a long time, and serious mental health problems are inevitable [19], which are often an important source of behavioral deviation. Therefore, to fill these research gaps, create a safe diagnosis and treatment environment and ensure the stability of the nursing team, it is important to investigate the workplace deviant behavior and mechanism of new nurses.

## Background

In previous studies, perceived organizational justice is an important antecedent variable affecting workplace deviant behavior [20, 21]. The perception of perceived organizational justice refers to the subjective feeling of organization members on whether the distribution, information and interpersonal relationships of the organization are fair [22]. A good sense of organizational justice can enhance professional identity [23], affect the work performance of nurses, and have an important impact on individual work development [24]. Social cognitive theory is an important theory in organizational behavior. This theory emphasizes that an individual’s behavior is influenced by the situation they are in, and information is integrated through their internal cognition to exhibit corresponding behaviors [25]. Perceived organizational justice is an important situational factor in an organization, which is an individual’s perception of the organization and can have a significant impact on their behavior. Kakemam [26] found that perceived organizational justice is negatively correlated with workplace deviant behavior, and significantly negatively predicts workplace deviant behavior, which is consistent with the previous study [27]. At the same time, study has proved that information justice and work atmosphere can affect workplace deviant behavior [28]. In order to better understand the perceived organizational justice perception and workplace deviant behavior of new nurses, we proposed the following hypothesis:

### H1

 The perceived organizational justice of new nurses is negatively correlated with workplace deviant behavior.

Emotional labor has attracted more and more attention among scholars [29, 30]. Emotional labor refers to that nurses can reasonably adjust and express their internal and external emotions according to the requirements of their work roles when facing patients [31]. Appropriate emotional labor can improve nurses’ strain capacity, but a high level of emotional labor will lead to job burnout, depression and other adverse emotions, and also affect patients’ satisfaction with nursing services [32]. We use conservation of resources theory to reveal the underlying mechanism by which perceived organizational justice affects workplace deviant behavior. The conservation of resources theory includes the spiral principles of resource loss and the spiral principles of resource acquisition [33]. The former refers to individuals constantly consuming resources when facing pressure, and when they are depleted, they will take action to prevent the continuous loss of resources. The latter is when individuals have more resources, they will choose to acquire more resources to prevent the loss of resources. When nurses experience less sense of justice in the organization, they will adopt emotional labor strategies to cope. Emotional labor, as a new form of labor after physical strength and intelligence [30], is a source of stress. According to the spiral principle of resource loss in the conservation of resources theory, emotional labor constantly consumes psychological resources and thus produces emotional exhaustion [34], reduces the happiness of work [35], and affects work behavior and work state [36]. In order to compensate for the loss of resources, nurses may adopt negative behaviors, which can lead to workplace deviant behavior. Furthermore, a study of medical personnel showed that emotional labor can positively predict the level of deviant behavior [37]. Therefore, this study proposes the following hypothesis:

### H2

 Emotional labor plays a mediating role in the relationship between perceived organizational justice and workplace deviant behavior of new nurses.

Psychological capital is a positive mental state owned by an individual and a potentially available internal resource of an individual, which can help an individual cope with difficulties and setbacks [38]. It can weaken the adverse effects of negative emotions, and promote the growth and development of an individual [38]. Studies have pointed out that individual behavior can be influenced by four dimensions of psychological capital (hope, self-efficacy, optimism and resilience) [39]. Based on the spiral principle of resource acquisition in the conservation of resources theory, people will establish, protect and cultivate their own resource base [33]. If individuals have enough psychological resources to supplement and cope with resource consumption, the adverse consequences brought by pressure will be effectively alleviated or eliminated [40]. When emotional labor consumes resources, psychological capital, as a resource supplement, can reduce the level of emotional labor [41], which can improve the mental health of nurses and alleviate the appearance of workplace deviant behavior. Previous studies have pointed out that emotional labor is correlated with psychological capital [42]. Kim [34] found that positive psychological capital and social support of special education teachers can regulate the impact of emotional labor on job burnout, and thus affect career happiness. Other studies have found that psychological capital and psychosocial safety atmosphere can improve the positive service behaviors of service workers towards work and customers [43], which promotes occupational health and safety. Therefore, the following hypothesis is proposed in this study:

### H3

 Psychological capital moderates the effect of emotional labor on workplace deviant behavior of new nurses.

**Fig. 1:**
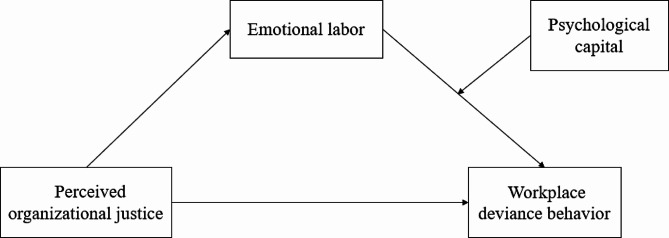
The theoretical framework model of this study

Previous studies have shown that perceived organizational justice is related to workplace deviant behavior of nurses, but the effects of emotional labor and psychological capital on perceived organizational justice and workplace deviant behavior have not been deeply explored. Therefore, the purpose of this study is to explore the mediating effect of emotional labor on perceived organizational justice and workplace deviant behavior, as well as the moderating effect of psychological capital, in order to provide a theoretical and practical basis for reducing workplace deviant behavior of new nurses and improving clinical nursing quality. The research framework is shown in Fig. 1.

## Method

### Participants and procedures

In this study, a cross-sectional study was used to investigate 5 hospitals in Henan Province, China from February to April 2023. Inclusion criteria: (1) registered nurses; (2) the working time is less than 1 year; (3) voluntary participation in this study. Exclusion criteria: nurses who go to the hospital for further study. A total of 574 questionnaires were collected for the study. In the process of questionnaire screening, the randomly filled questionnaires and all the questionnaires with the same answers were strictly excluded, and 546 valid questionnaires were obtained in the end.

### Measures

#### Demographic characteristics

Demographic information for this study included gender, age, education level, job department, labor relations, working hours per day, and monthly income.

#### Emotional labor

The Emotional labor Scale translated by Luo [44] was adopted to investigate the emotional labor of nurses. It consists of three dimensions: surface acting (7 items), emotional expression (4 items), and deep acting (3 items). One of the items is “In order to serve patients in an appropriate way, I learned to disguise my emotions”. The Cronbach’α of the scale in this study was 0.94.

#### Workplace deviant behavior

The workplace deviant behavior scale was developed by Bennett [8], and Zhang [9] was translated. There were 21 items in the scale, including behavioral deviation at the interpersonal level (6 items), behavioral deviation at the organizational level (6 items), aggressive behavior toward the service object (2 items), aggressive behavior toward organization members (4 items), and behavior violating labor discipline (3 items). For example, one of the items is “Deliberately concealing some information that others should know”. Cronbach’α of this scale is 0.98.

#### Perceived organizational justice

The survey of nurse’s perceived organizational justice was compiled by Colquitt [45] and translated by Zhang [46]. The scale consists of 20 items and four dimensions, including distributive justice (4 items), procedural justice (7 items), interpersonal justice (4 items), and information justice (5 items). For example, one of the items is “Leaders respect me”. Cronbach’α of this scale is 0.95.

#### Psychological capital

The psychological capital of nurses was measured using the psychological capital questionnaire (PCQ) developed by Luthan [47]. Luo [48] was revised according to the characteristics of nursing work in China. The scale consisted of 20 items and was used to test four dimensions of self-efficacy (6 items), hope (6 items), resilience (5 items) and optimism (3 items). For example, one of the items is “I am able to achieve my work goals with full energy.” Cronbach’s α of the questionnaire ranged from 0.72 to 0.92. The Cronbach’s α of this scale in this study was 0.97.

#### Ethics considerations

The researcher explains the purpose of the scientific study to the hospital manager and obtains permission for the investigation. At the same time, after learning the purpose of the study on the first page of the questionnaire, the respondents voluntarily participated in the study and could withdraw from the survey at any time. The content of the study did not harm the participants’ physical and mental health. Results were used for this study only and non-researchers were not authorized to view the questionnaires collected. This study was approved by the Ethics Committee of Henan University, and the ethics review number is HUSOM2022-375.

### Data analysis

SPSS 26.0 and PROCESS Macro were used for data analysis. Confirmatory factor analysis was conducted using AMOS 28. Descriptive statistical methods are used to describe data frequency, mean, standard difference, etc. Pearson correlation analysis was used to verify the correlation among perceived organizational justice, emotional labor, psychological capital and workplace deviant behavior. The influence of demographic data on workplace deviant behavior is analyzed by multiple linear regression. Then, the mediating effect of emotional labor was tested using PROCESS Model 4 developed by Hayes [49]. Finally, Model 14 was used to verify the moderating effect of psychological capital. We used the 5000 resample bootstrapping method with a 95% CI to test the model. When 95% CI does not include 0, the effect is meaningful. All analyses were two-tailed, and the level of statistical significance was *p* < 0.05.

## Result

### Descriptive statistics of population data

The results of the demographic analysis of nurses in this study showed that the majority of nurses were female (72.53%) and had an undergraduate degree (66.85%). 69.78% work in tertiary hospitals. 63.55% are contract workers. New nurses with monthly income below 4000 RMB were more than half (64.28%). The detailed results are shown in Table 1.

**Table 1:** Demographic characteristics(*N* = 546)

Variables		n	%
Gender	Male	150	27.47
	Female	396	72.53
Record of formal schooling	Junior college and below	101	18.50
	Undergraduate Degree	365	66.85
	Master degree or above	80	14.65
Class of hospital	Level III hospital	381	69.78
	Level II and below hospitals	165	30.22
Administrative office	Internal medicine department	166	30.40
	Surgery department	113	20.70
	Gynecology and obstetrics	40	7.33
	Pediatric department	33	6.04
	Emergency department	39	7.14
	Intensive care unit	27	4.95
	Others	128	23.44
Labor and personnel relations	Regular establishment staff	102	18.68
	Personnel agency	97	17.77
	Contract worker	347	63.55
Average monthly income(RMB)	< 4000	351	64.28
	4000 ~ 7000	153	28.02
	7001 ~ 10,000 RMB	21	3.85
	>10,000 RMB	21	3.85

### Correlation analysis

The mean value, standard deviation of the study variables and the correlation among variables are shown in Table 2. The item’s mean scores of perceived organizational justice, emotional labor, psychological capital, and workplace deviant behavior were 3.61 ± 0.75, 3.16 ± 0.90, 4.64 ± 0.83, 1.67 ± 0.98, respectively. Perceived organizational justice and workplace deviant behavior (*r*=-0.40, *p* < 0.01) were negatively correlated, which validates hypothesis 1. Emotional labor was negatively correlated with perceived organizational justice (*r*=-0.46, *p* < 0.01), but positively correlated with workplace deviant behavior (*r* = 0.40, *p* < 0.01).

**Table 2:** Descriptive statistics and correlation analysis of variables(*N* = 546)

Variables	CR	AVE	1	2	3	4	Mean ± SD
1. Perceived organizational justice	0.95	0.66	**0.81**				3.61 ± 0.75
2. Emotional labor	0.93	0.59	-0.46^**^	**0.77**			3.16 ± 0.90
3. Psychological capital	0.92	0.57	0.60^**^	-0.39^**^	**0.76**		4.64 ± 0.83
4. Workplace deviant behavior	0.97	0.79	-0.40^**^	0.40^**^	-0.41^**^	**0.89**	1.67 ± 0.98

Note: CR: Composite reliability, AVE: Average variance extracted, SD: Standard deviation. The Diagonal is the square root of AVE. ^**^p < 0.01

### Confirmatory factor analysis of measurement models

The average variance extracted (AVE) and composite reliability (CR) of the study variables were both higher than the threshold (AVE > 0.5, CR > 0.7) [50] (Table 2), indicating good internal consistency and reliability of the study measurement model. In addition, collinearity diagnosis shows that the variance inflation factor (VIF) is less than 5, indicating that there is no collinearity issue between variables [51].

To further validate the structural validity of the research variables, we conducted a confirmatory factor analysis using AMOS 28 on perceived organizational justice, emotional labor, psychological capital, and workplace deviant behavior, then compared the hypothesized four factors model with three other possible models (Table 3). The results showed that the four factors model had a better fit (*x*^2^**/***df* = 2.755, CFI = 0.986, IFI = 0.986, RMSEA = 0.057). This result supports the uniqueness of the model proposed in this study.

**Table 3:** Confirmatory factor analysis

Models	x^2^/df	RMSEA	IFI	CFI
Hypothesized four-factor model	2.755^***^	0.057	0.986	0.986
Three-factor model (combining WDB and EL)	3.339^***^	0.066	0.982	0.982
Two-factor model (combining WDB and EL, PC)	5.241^***^	0.088	0.971	0.971
Single-factor model (combining WDB, EL, PC, POJ)	16.087^***^	0.166	0.875	0.875

Note: WDB: Workplace deviant behavior, EL: Emotional labor; PC: Psychological capital, POJ: Perceived organizational justice, *χ*^2^: chi-square; *df*: Degree of freedom, RMSEA: Root Mean Square Error of Approximation, IFI: Incremental Fit Index, CFI: Comparative Fit Index. ^***^p < 0.001

### Perceived organizational justice and workplace deviant behavior: a moderated mediation test

The results of multiple linear regression analysis of demographic data show that the average monthly income has a significant effect on workplace deviant behavior. Therefore, average monthly income was analyzed as a control variable.

Firstly, Model 4 in the SPSS PROCESS was used to test the mediating effect of emotional labor. The results are shown in Table 4. Perceived organizational justice had a significant negative predictive effect on workplace deviant behavior (c=-0.52, 95% CI: -0.63 to -0.42). Emotional labor had a significant mediating effect between perceived organizational justice and workplace deviant behavior, and the effect value was − 0.17 (95% CI: -0.23 to -0.12), accounting for 32.7% of the total effect. When perceived organizational justice and emotional labor entered the regression equation together, the predictive effect of perceived organizational justice on workplace deviant behavior was still significant (c’ =-0.35, 95% CI: -0.46 to -0.24). Therefore, emotional labor plays a partial mediating role in the prediction of perceived organizational justice on workplace deviant behavior of new nurses, which validates hypothesis 2.

Secondly, Model 14 in the SPSS PROCESS was used to test the moderating effect of psychological capital, and the test results are shown in Table 5. Perceived organizational justice significantly negatively predicted workplace deviant behavior (*β* = 0.52, *p* < 0.001). Perceived organizational justice significantly negatively predicted emotional labor (*β*=-0.56, *p* < 0.001). Then, the main effect of emotional labor on workplace deviant behavior was significant (*β* = 0.26, *p* < 0.001), and the interaction terms of perceived organizational justice and emotional labor predicted workplace deviant behavior significantly (*β*=-0.23, *p* < 0.001).

**Fig. 2:**
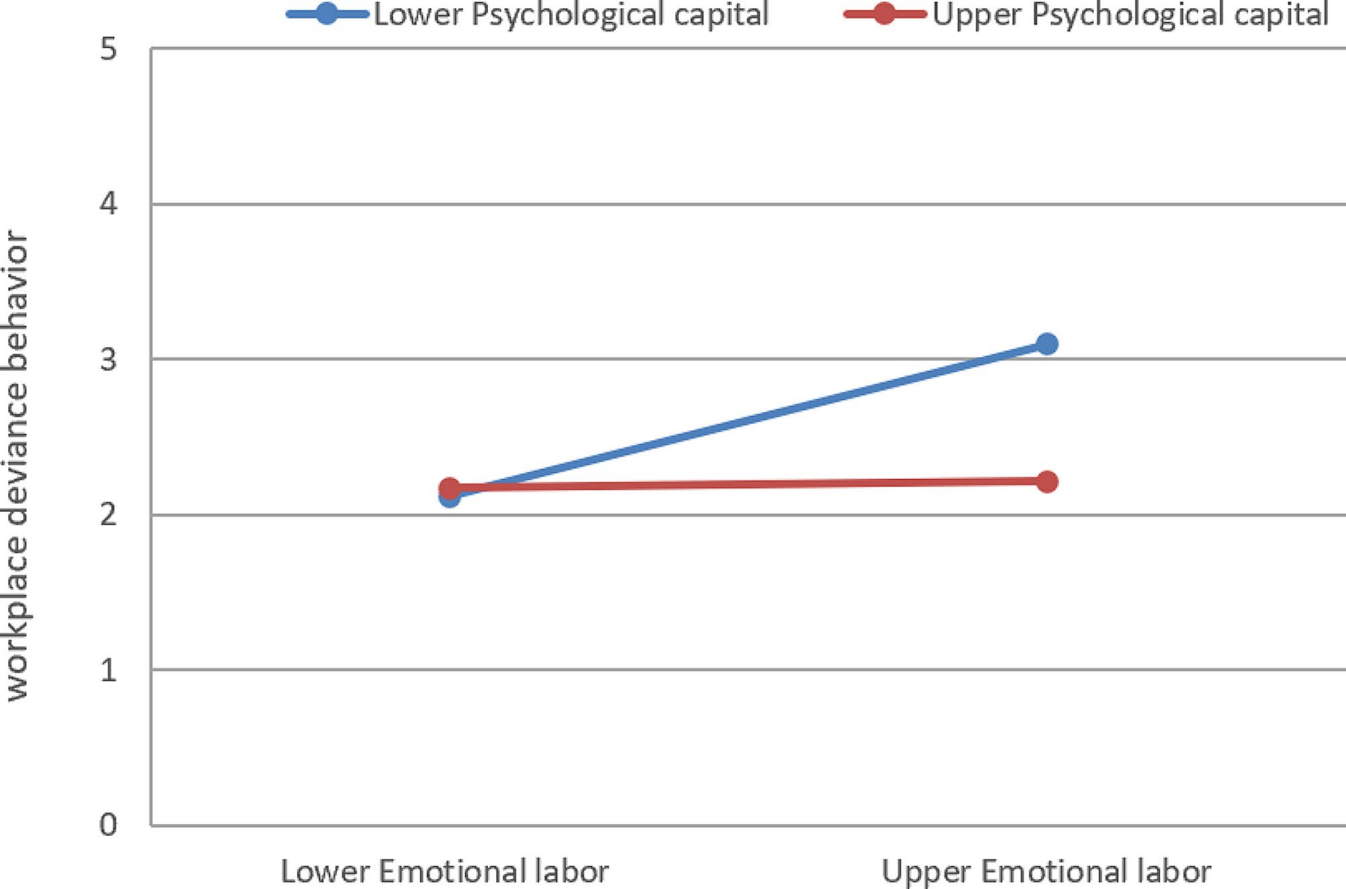
The moderating effect of psychological capital

Further simple slope analysis results are shown in Fig. 2. For new nurses with low psychological capital, emotional labor had a significant positive predictive effect on workplace deviant behavior (*β*_simple slope_ = 0.46, 95% CI: 0.34 to 0.58), but for new nurses with high psychological capital, emotional labor had no significant positive predictive effect on workplace deviant behavior (*β*_simple slope_ = 0.07, 95% CI: -0.06 to 0.19). The results showed that emotional labor had different predictive effects on the deviation behavior of new nurses under different levels of psychological capital, which validates hypothesis 3. To be specific, emotional labor has a more significant positive predictive effect on workplace deviant behavior when psychological capital is low.

**Table 4:** Total, direct, and indirect effects of perceived organizational justice on workplace deviant behavior(*N* = 546)

Effects	Paths	Effect	SE	Bootstrapping 95% CI	*p*
Total effect	POJ→WDB	-0.52	0.05	-0.63 to -0.42	<0.001
Direct effect	POJ→WDB	-0.35	0.06	-0.46 to -0.24	<0.001
Indirect effect	POJ→EL→WDB	-0.17	0.03	-0.23 to -0.12	<0.001

Note: POJ: Perceived organizational justice, EL: Emotional labor, WDB: Workplace deviant behavior, SE: Standard error, CI: Confidence interval

**Table 5:** The model of mediating effect with moderating

Predictive variable	Model 1(Outcome variable WDB)			Model 2(Outcome variable EL)			Model 3(Outcome variable WDB)	
	β	t		β	t		β	t
Average monthly income	0.05	0.93		-0.10	-2.13^*^		-0.08	-1.69
POJ	-0.52	-10.16^***^		-0.56	-12.31^***^		-0.21	-3.29^***^
EL							0.26	5.72^***^
PC							-0.21	-3.75^***^
EL*PC							-0.23	-4.58^***^
R^2^	0.16			0.22			0.28	
F	53.23^***^			76.31^***^			42.69^***^	

Note: POJ: Perceived organizational justice, EL: Emotional labor, PC: Psychological capital, WDB: Workplace deviant behavior, ^*^p < 0.05, ^**^p < 0.01, ^***^p < 0.001

## Discussion

This study constructed a moderated mediation model to explore the mechanism of the relationship between perceived organizational justice and workplace deviant behavior of new nurses. On the one hand, it illustrates that perceived organizational justice affects the workplace deviant behavior of new nurses through the mediating effect of emotional labor. On the other hand, it explains that psychological capital can regulate the latter half path of intermediary (the influence of emotional labor on workplace deviant behavior). Compared with new nurses with low psychological capital, high psychological capital weakens the influence of emotional labor on workplace deviant behavior. The results of this study provide theoretical and practical significance for the prevention and intervention of work behavior problems of new nurses.

In this study, the perceived organizational justice score is slightly lower than that of Viseu [52], and the difference in results may be due to the difference in the research objects. In the process of changing from the school environment to the hospital working environment, the complex working environment and interpersonal relationships make the new nurses have a sense of unevenness in the fairness of the hospital organization. The score of emotional labor was similar to that of Yu [53], indicating that there was a certain level of emotional labor among new nurses. On the one hand, new nurses are still adapting to hospital work, on the other hand, they are faced with an overloaded workload and lack coping skills in dealing with stressful events and negative emotions, so emotional labor may occur. In addition, the level of psychological capital is higher than the previous results of Kim [54], which may be due to the difference in sample size. Finally, the workplace deviant behavior score was higher than Zhang [28], indicating that the nursing quality and organizational discipline of new nurses still need to be improved. The reasons may be related to the less experience of new nurses and the lack of predictability and sensitivity to clinical emergencies, which has an impact on the results of work quality.

This study confirmed that perceived organizational justice negatively predicted workplace deviant behavior, indicating that perceived organizational justice was a protective factor for workplace deviant behavior of new nurses, which was consistent with Kakemam’s [26] research. According to social exchange theory, individuals will evaluate the potential costs and benefits to obtain the best benefit result, and then choose to give back to the organization or help themselves [55]. Nurses regard their relationship with the organization as a social exchange. When nurses feel fair treatment, they will actively exchange with the hospital, which is reflected in the improvement of work performance [23]. However, when nurses feel unfair, they will destroy the social exchange and take deviant behaviors to reduce their own losses [56, 57]. Therefore, the improvement of organizational justice can reduce deviant behavior. Nursing managers should create a fair organizational environment for new nurses. In the nursing process and the implementation of decision-making control process, managers should pay attention to procedural equity, interpersonal equity, information equity, and distribution equity, so as to make nursing management work more just and transparent, reducing the occurrence of deviant behavior.

This study also found that emotional labor played a mediating role in the relationship between perceived organizational justice and workplace deviant behavior of new nurses. When comparing the new nurses with the surrounding senior nurses and doctors, it is found that there is a big gap in salary and welfare treatment, and nurses feel unfair, which leads them to feel that their labor has not been equally supplemented [58], resulting in a feeling of injustice. Low perceived organizational justice reduces nurses’ sense of collective belonging, resulting in higher emotional labor [59]. High emotional labor will lead to emotional exhaustion, job burnout, turnover intention, etc [60, 61]., which promote deviant behavior [62]. At the same time, based on the psychological contract theory [63], when employees plan to leave the organization, that is, when the turnover intention is high, the employees may not firmly abide by the standard requirements of the organization. A large number of studies have shown that emotional labor will lead to high turnover intention of nurses [64, 65], resulting in a series of withdrawal behaviors, work deviation and lower work performance of nurses [66]. Therefore, nursing managers should reasonably formulate the salary performance appraisal system, reasonably arrange work schedules, pay attention to the emotional changes of new nurses and identify the emotional labor level of nurses as early as possible, and at the same time conduct regular psychological and emotional guidance for new nurses, which is beneficial to reduce the turnover rate of new nurses and improve the quality of work.

This study further found that the path that perceived organizational justice affects the workplace deviant behavior of new nurses through emotional labor is moderated by psychological capital. Specifically, the emotional labor of new nurses with low psychological capital has a stronger predictive effect on workplace deviant behavior, while for new nurses with high psychological capital, the predictive effect is weaker. The research results supported the conservation of resources theory [40]. In order to alleviate the impact of emotional labor pressure, individuals will mobilize resources to cope. However, for those with poor resources, resource input will not make ends meet, resulting in resource loss, which may lead to deviant behavior [67]. As an important psychological resource, nurses with high psychological capital have positive psychological qualities such as self-efficacy, hope, resilience and optimism, which provide mental and psychological support for nurses, relieve work pressure [68], and reduce the harm of resource consumption. At the same time, new nurses with high psychological capital have clear work goals and plans, and they can adapt to the changes of clinical work, quickly integrate into the work environment, and successfully become professional nursing staff [69], reducing deviant behavior. This suggests that managers should pay attention to the mechanism of psychological capital on workplace deviant behavior. They can create a supportive organizational atmosphere to improve their positive psychological capital, such as providing care and support for medical staff from the organization and carrying out corresponding psychological training courses.

### Relevance to clinical practice

New nurses are the young blood of the nursing team and have the potential for career development. However, new nurses have to face many pressures such as work, role and interpersonal relationships after entry. Forced management and restriction will cause a decrease in work efficiency and an increase in the turnover rate of new nurses [70], resulting in workplace deviant behavior. However, there is a great need for a stable nursing team to provide high-quality nursing services. Therefore, it is very important to reduce the deviation behavior of new nurses. This study provides a theoretical and practical basis for reducing the workplace deviant behavior of new nurses. In order to reduce workplace deviant behavior, first of all, nursing managers should give appropriate authorization at work to build an effective communication platform and opportunities for nurses to participate in decision-making, so that nurses can dare to express their ideas and communicate with managers actively when they feel unfair. At the same time, they should make full use of intelligent stimulation and contingent rewards to improve nurses’ sense of belonging to a team and happiness. Secondly, nursing managers should actively create a united and relaxed working atmosphere to reduce nurse patient conflicts. At the same time, reasonable work schedules should be formulated to reduce work intensity and reduce the emotional labor of new nurses, so that nurses can engage in work with positive emotions and improve the workplace deviant behavior of clinical nurses. Thirdly, nursing managers should pay attention to the psychological capital of new nurses. They can organize collective activities, build a support system, improve the psychological capital of new nurses, and effectively reduce workplace deviant behavior. Finally, the government plays an indispensable role in healthcare. The government can strengthen the supervision of medical institutions, establish clear nursing standards, and optimize the working environment of hospitals. At the same time, the government should attach importance to the psychological education of nurses and offer relevant courses on psychological education, so that newly graduated nurses have good psychological qualities to enter clinical work.

### Limitations and future research directions

There are some limitations to this study. First of all, this study was only conducted in Henan Province, which will limit the popularization of the research results. It is suggested that future multi-regional hospital studies should be carried out to make the findings more universal. Secondly, this study was cross-sectional and cannot determine causation. All subsequent studies could conduct longitudinal studies to further explore the links between the variables. Finally, this study used self-reporting to measure variables, and nurses may not fully express their true answers in order to present an ideal social image. Future studies could use more objective measurement methods, such as experimental intervention studies.

## Conclusion

In this study, the results showed that emotional labor played a mediating role between perceived organizational justice and workplace deviant behavior of new nurses, and psychological capital moderated the relationship between emotional labor and workplace deviant behavior. Therefore, we suggest that nursing managers on the one hand can guide new nurses to conduct emotional counseling and reasonable management of emotional labor. On the other hand, some support resources can be provided for new nurses, such as reward mechanisms, good doctor-patient relationship maintenance, and nurses can be encouraged to set goals and motivate work to improve the psychological capital level of new nurses. Under the joint action of reducing emotional labor and improving psychological capital, the work behavior of new nurses can be improved to create safe and high-quality nursing services.

## Data Availability

The data for this study is not publicly available. But it can be obtained from the author on reasonable demand.

## Abbreviations

SPSS: Statistical Product and Service Solutions
COVID-19: Corona Virus Disease 2019
